# Predictive factors for the detection of occult metastases during staging laparoscopy in patients with gastric carcinoma and adenocarcinoma of the esophagogastric junction

**DOI:** 10.1007/s00423-025-03783-9

**Published:** 2025-07-04

**Authors:** Felix von Bechtolsheim, Mareike Spindler, Veith Jungmann, Florian Oehme, Jürgen Weitz, Thilo Welsch, Benjamin Müssle

**Affiliations:** 1 Department of Visceral-, Thoracic and Vascular Surgery, Faculty of Medicine and University Hospital Carl Gustav Carus, TUD Dresden University of Technology, Fetscherstraße 74, 01307 Dresden, Germany; 2 Department of General, Visceral and Thoracic Surgery, University Hospital Hamburg-Eppendorf, Hamburg, Germany; 3 Department of General and Visceral Surgery, University Hospital Ulm, Ulm, Germany; 4 Surgical Skills Lab Dresden, Medical Faculty and University Hospital Carl Gustav Carus, TUD Dresden University of Technology, Fetscherstraße 74, 01307 Dresden, Germany

**Keywords:** Staging laparoscopy, Adenocarcinoma of the esophagogastric junction, Gastric cancer, Staging, Occult metastasis

## Abstract

**Introduction:**

Peritoneal metastasis can occur in all stages of gastric cancer (GC) and adenocarcinoma of the esophagogastric junction (AEG) but staging laparoscopy (SL) is recommended for advanced stages. This study aimed to evaluate predictive factors for the detection of further, previously unknown (occult) metastases during SL.

**Materials & methods:**

We conducted a retrospective analysis of patients who underwent SL at our center between 2005 and 2018. Binary logistic regression analysis was used to identify risk factors for the occurrence of occult metastasis.

**Results:**

A total of 232 patients were included in the analysis. Occult metastases were detected in 48 (20.7%) patients. Forty patients (17.2%) had peritoneal carcinomatosis, 4 (1.6%) had liver metastases, 3 (1.2%) had peritoneal and liver metastases, and 1 (0.4%) had omental metastases. Univariate analysis revealed that cT4 category; cM-positivity; WHO G3 grade; histology results revealing diffuse, mixed or undifferentiated Lauren subtypes; and signet ring cells were significant risk factors for occult metastasis. Multivariate analysis confirmed that cM-positive stage (OR: 17.672; 95% CI: 3.06 to 102.052; *p* = 0.001) and signet ring cell count (OR: 6.228; 95% CI: 1.151 to 33.716; *p* = 0.034) were independently associated with occult metastasis detection by SL.

**Conclusion:**

Occult metastases are common in patients with GC or AEG who undergo SL. Histological evidence of signet ring cells should be considered a high-risk histology result and should be an independent indication for SL. Patients with positive cM staging might benefit from SL because of the high probability of further occult metastases.

**Supplementary Information:**

The online version contains supplementary material available at 10.1007/s00423-025-03783-9.

## Introduction

Gastric cancer (GC) and adenocarcinoma of the esophagogastric junction (AEG) remain some of the most prevalent cancers worldwide. Curative treatment for GC and AEG consists of surgical resection combined with perioperative chemotherapy [1–3]. Unfortunately, despite advances in surgical techniques, and perioperative management as well as increasing centralization at high-volume centers, the prognosis of patients who receive curative treatment remains poor, and the 5-year overall survival rate of these patients is 20–40%. The main reason for this poor prognosis is local recurrence [1, 4]. Peritoneal carcinomatosis is the most common form of metastatic disease in patients with CG and AEG. The presence of peritoneal carcinomatosis is associated with a higher risk of mortality and disease progression and ultimately with poorer survival rates [5–7]. 

The standard procedure for diagnosing these patients involves gastroscopy to determine the tumor size and to collect tissue samples for the histopathological characterization of the tumor; furthermore, computed tomography (CT) of the thorax and abdomen is used to detect metastases and determine local resectability [8]. Computed tomography is used for further environmental diagnosis as part of the staging process. The accuracy of CT for detecting metastatic disease or locally advanced disease ranges between 72% and 86% [9, 10]. The diagnosis of peritoneal metastases by CT often depends on the detection of unique features such as ascites, omental cake and peritoneal thickening, but smaller deposits (< 5 mm), such as peritoneal nodules, may often be missed [11, 12]. The overall sensitivity and specificity of peritoneal nodule detection ranges between 75% and 92%, respectively [9]. 

Staging laparoscopy (SL) is therefore recommended to exclude the possibility of peritoneal metastasis before resection especially for patients with advanced stage tumors [13, 14]. Although SL may not have additional benefits for detecting lymph node metastasis, it has high sensitivity for detecting peritoneal carcinomatosis and for estimating and quantifying the overall extent of abdominal metastasis, with the exception of deep liver metastases [15]. 

In particular, peritoneal carcinomatosis frequently occurs as occult metastases, i.e., as a tumor manifestation that is not diagnosed during staging studies and is identified only by SL. Advanced tumor stages of GC and AEG are often associated with peritoneal carcinomatosis and further distant metastases. However, patients with early-stage tumors may also exhibit peritoneal tumor spread. According to the literature, peritoneal carcinomatosis has been reported in 1% and up to 13% of patients with T1 and T2 tumors, respectively [16, 17]. 

Radiographic underestimation of tumor staging may lead to unsuccessful surgical exploration. In these cases, SL may not only help to avoid unsuccessful surgical exploration but also support decision-making about neoadjuvant therapy. As recent publications suggest, combinations of neoadjuvant chemotherapy with cytoreductive surgery and hyperthermic intraperitoneal chemotherapy (HIPEC) might be promising therapeutic approaches for patients with metastatic GC and AEG [18–21]. However, these patients should be carefully selected based on accurate staging [20, 22]. 

Although the use of SL prior to surgical exploration is widely described and recommended, the indication for SL in some patients with GC and AEG remains unclear, particularly in patients with early-stage tumors. Therefore, the aim of the present study was to evaluate the clinical value of SL. Furthermore, we aimed to identify predictive factors for the occurrence of occult metastases as detected by SL and their impact on the further treatment and clinical management of patients with GC or AEG.

## Materials and methods

### Patient cohort

**Fig. 1:**
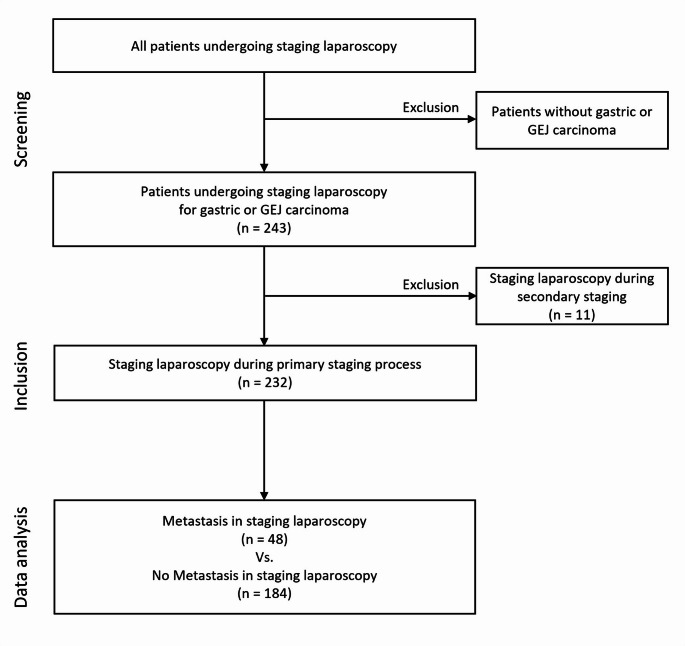
Schematic trial design

For this retrospective study, the data of 243 consecutive patients who underwent SL due to GC or AEG were analyzed. According to the standard operating procedure, the indication for SL was a suspected staging of T3 or higher, suspected lymph node infiltration or ascites (Fig. 1). In addition, extended indication for SL based on clinical appearance and markers, other staging diagnostics or recommendations of the interdisciplinary tumor board. These extended indications may include clarifying suspected metastases on imaging or assessing their extent. While the risk of peritoneal metastasis in clinical T1-T2 cases is low, SL may still be performed in selected cases with concerning findings to ensure accurate staging. SL was conducted between March 2005 and October 2018 at the Department of Visceral, Thoracic and Vascular Surgery, University Hospital Carl Gustav Carus, TU Dresden. This study was approved by the local ethics committee (decision no. BO-EK-546122020).

All the clinical data, including patient demographics (age and sex), habits (tobacco and alcohol consumption), weight, height, body mass index (BMI), American Society of Anesthesiologists (ASA) score, comorbidities, radiographic and endoscopic diagnostics and tumor-specific data (histology, location and staging), were obtained retrospectively.

Only patients who did not receive neoadjuvant therapy were included, therefore eleven patients were excluded from further analysis because laparoscopy was initiated as a restaging diagnostic approach after previous therapy. Therefore, only 232 patients were included in the final statistical analysis.

### Primary endpoint

Occult metastasis includes all metastases detected or newly diagnosed during SL that had not yet been detected on previous staging diagnostic procedures. This means that patients who had previously been diagnosed with metastasis may also have had additional occult metastasis.

### Staging laparoscopy

The patients were placed in a supine position under general anesthesia. A 10-mm periumbilical port was inserted into the abdominal cavity, and a pneumoperitoneum was established with CO_2_ under 15 mmHg pressure. Laparoscopy was performed using a 30° telescope. First, the 4 quadrants of the abdominal cavity were thoroughly explored. Additional trocars were inserted into the abdominal cavity according to the preferences of the primary surgeon to allow for the use of grasping or biopsy forceps. A detailed exploration of the liver, stomach, small and large intestine, lesser sac, diaphragm, serosal surfaces, peritoneum, omentum and pelvic organs was systematically performed. Biopsies were taken from suspicious cancerous lesions and sent for histopathological examination. The locations of all suspicious lesions were documented during the surgical protocol.

### Statistical analysis

Statistical data analysis was performed using the IBM SPSS Statistics program (version 23, SPSS, Inc., Chicago, IL, USA). The values are expressed as medians and interquartile ranges (IQRs) unless otherwise indicated. A p value less than 0.05 indicated statistical significance. All the variables with a significant p value of < 0.05 in the univariate analysis were subjected to multivariate analysis to identify independent risk or protective factors. The odds ratios for independent variables regarding the occurrence of further, occult metastasis in all 232 patients were analyzed using a binary logistic regression model.

## Results

### Basic patient characteristics

During the observation period, 232 patients underwent SL as a part of the routine staging diagnostic procedure for diagnosed GC or AEG. Depending on the SL outcome, treatment was continued in curative or palliative intent. The majority of patients were male (*n* = 161; 69.4%). The median age was 75 years (IQR 66–83). The median BMI was 25 kg/m^2^ (IQR 22.8–28.1). Approximately half of the patients neither smoked (52.2%) nor consumed alcohol (50%). More than half of the patients (57.8%) had an American Society of Anesthesiologists (ASA) grade of 2. Comorbidities were prevalent in almost all patients (87.9%). The most frequent comorbidity was hypertension (50%). Most of the tumors were located in the gastric corpus (26.3%) or at the esophagogastric junction (24.1%) (Table 1).

**Table 1:** Patient characteristics (after exclusion of 11 patients who underwent SL during the restaging process)

Basic patient characteristics			
Age [years] (IQR)			75 (66–83)
Sex [n] (%)		male	161 (69.4)
		female	71 (30.6)
ASA [n] (%)		1	20 (8.6)
		2	134 (57.8)
		3	67 (28.9)
		missing	11 (4.7)
Height [m] (IQR)			1.7 (1.7–1.8)
Weight [kg] (IQR)			74 (65–85)
BMI [kg/m ^2^ ] (IQR)			25 (22.8–28.1)
Tobacco use [n] (%)		never smoked	121 (52.2)
		previous smoker	34 (14.7)
		current smoker	60 (25.9)
		missing	17 (7.3)
Alcohol consumption [n] (%)		no alcohol	116 (50)
		occasionally	35 (15.1)
		daily	60 (25.9)
		missing	21 (9.1)
Comorbidities [n] (%)		COPD	10 (4.3)
		Renal failure	17 (7.3)
		Congestive heart failure	9 (3.9)
		Hypertension	116 (50)
		Cerebrovascular disease	8 (3.4)
		Coronary artery disease	26 (11.2)
		Diabetes	53 (22.8)
		Deep vein thrombosis	10 (4.3)
Previous abdominal surgery [n] (%)			66 (28.4)
Tumor location [n] (%)		AEG I	28 (12.1)
		AEG II	56 (24.1)
		AEG III	15 (6.5)
		AEG II-III	2 (0.9)
		Proximal/Fundus	2 (0.9)
		Antral	42 (18.1)
		Corpus	61 (26.3)
		Pylorus	2 (0.9)
		diffuse	23 (9.9)
		missing	1 (0.4)
Diagnostics [n] (%)		No initial diagnostics	1 (0.4)
		Endoscopy	227 (97.8)
		CT	201 (86.6)
		MRI	13 (5.6)
		PET	60 (25.9)
		Endosonography	141 (60.8)
		Scintigraphy	3 (1.3)
		X-ray	97 (41.8)
		Sonography	99 (42.7)
		Contrast swallow	45 (19.4)
CT-Staging [n] (%)		cT1	3 (1.3)
		cT2	19 (8.2)
		cT3	159 (68.5)
		cT4	24 (10.3)
		missing	27 (11.6)
cN-Status [n] (%)		cN-	44 (19)
		cN+	165 (71.1)
		missing	23 (9.9)
cM-Status [n] (%)		cM-	135 (58.2)
		cM+	33 (14.2)
		missing	64 (27.6)
EUS Staging [n] (%)		T1:	3 (1.3)
		T2:	12 (5.2)
		T3:	94 (40.5)
		T4:	16 (6.9)
		missing	107 (46.1)
EUS N-Status [n] (%)		N-	53 (22.8)
		N+	55 (23.7)
		missing	124 (53.4)
WHO-grade [n] (%)		G1	7 (3)
		G2	54 (23.3)
		G3	125 (53.9)
		G2-G3	4 (1.7)
		missing	42 (18.1)
Barett carcinoma [n] (%)			12 (5.2)
Laurén classification [n] (%)		Intestinal	68 (29.3)
		diffuse	71 (30.6)
		mixed	11 (4.7)
		Undifferentiated	2 (0.9)
		missing	79 (34.1)
Signet ring cell [n] (%)			54 (23.3)
UICC [n] (%)		IA	1 (0.4)
		IB	4 (1.7)
		II	44 (19)
		IIIA	97 (41.8)
		IIIB	6 (2.6)
		IV	70 (30.2)
		missing	10 (4.3)
Time from diagnosis to laparoscopy [days] (IQR)			32.5 (23–45)
Incidence of occult metastasis [n] (%)			48 (20.7)
Localisation of occult metastasis [n] (%)			
	Peritoneum		40 (17.2)
	Liver		4 (1.7)
	Peritoneum + Liver		3 (1.3)
	Omentum		1 (0.4)

Abbreviations: *IQR *interquartile range, *ASA *American society of anesthesiologists, *BMI *body mass index, *COPD *chronic obstructive pulmonary disease, *AEG *adenocarcinoma of the esophagogastric junction, *CT *computed tomography, *MRI *magnetic resonance imaging, *PET* positron emission tomography, *EUS *endoscopic ultrasonography, *WHO *world health organization; UICC: union internationale Conte Le Cancer classification

### Preoperative staging diagnostics

Endoscopy (97.8%) and CT (86.6%) were performed for almost all the patients during the staging process. Endosonography was performed in 60.8% of patients, and transabdominal sonography was performed in 42.7% of patients.

According to the CT diagnosis, the tumors of 159 patients were staged as cT3 (68.5%). The tumors of twenty-four (10.3%) patients were classified as cT4, 19 (8.2%) were staged as cT2 and 3 (1.3%) were staged as cT1.

With CT staging, lymph node involvement (cN+) was suspected in 165 patients (71.1%), and distant metastases (cM+) were suspected in 33 patients (14.2%). In the patients receiving endosonography, the tumor of 3 (1.3%), 12 (5.2%), 94 (40.5%) and 16 (6.9%) patients were staged as T1, T2, T3 and T4, respectively. Here, 55 (23.7%) patients were diagnosed with lymphatic metastasis.

### Histopathology

Histopathological analysis revealed Barrett carcinoma in 12 patients (5.2%) and signet ring cells in 54 patients (23.3%). According to the Lauren classification, 68 patients (29.3%) had intestinal disease, 71 (30.6%) had diffuse disease, 11 (4.7%) had mixed disease, and 2 (0.9%) had the undifferentiated subtype. The WHO tumor grade was G1 in 7 (3.0%), G2 in 54 (23.3%), G2-3 in 4 (1.7%) and G3 in 125 (53.9%) patients. The tumor of one patient (0.4%) was staged as UICC IA, those of 4 patients (1.7%) were staged as UICC IB, those of 44 patients (19.0%) were staged as UICC II, those of 97 patients (41.8%) were staged as UICC IIIA, those of 6 patients (2.6%) were staged as UICC IIIB and those of 70 patients (30.2%) were staged as UICC IV.

The median time between initial diagnosis and SL was 32.5 days (IQR 23–45). During SL, 48 patients (20.7%) were newly diagnosed with occult metastases that had not been detected during the staging diagnosis performed up to that point. These occult metastases were localized on the peritoneum in 40 patients, in or on the liver in 4 patients, in or on the liver and on the peritoneum in 3 patients and on the omentum in one patient.

### Risk factors for the occurrence of occult metastasis

A total of 232 patients were included in the analysis (Table 2). In the SL cohort, 184 patients were found to have no evidence of occult metastasis, whereas occult metastasis was detected in 48 patients.

**Table 2:** Uni- and multivariate analyses for the detection of occult metastasis by SL (*p* values < 0.05 are marked in bold)

Variables		No occult metastasis	Occult metastasis	Univariate			Multivariate		
				OR	CI	*p*-value	OR	CI	*p*-value
Age [years] (IQR)		74 (66–83)	77 (65–84)	0.999	(0.973–1.026)	0.995			
Sex [n] (%)									
male		129 (70.1)	32 (66.7)	1.173	(0.595–2.31)	0.645			
female		55 (29.9)	16 (33.3)						
ASA [n] (%)									
1		14 (7.6)	6 (12.5)	1.838	(0.663–5.095)	0.242			
2		110 (59.8)	24 (50)	0.731	(0.375–1.424)	0.357			
3		53 (28.8)	14 (29.2)	1.092	(0.536–2.224)	0.809			
BMI [kg/m] (IQR)		25.0 (22.7–28.1)	25.1 (22.8–27.8)	0.995	(0.923–1.074)	0.904			
Tobacco use [n] (%)									
never smoked		93 (50.5)	28 (58.3)	1.876	(0.911–3.862)	0.088			
previous smoker		29 (15.8)	5 (10.4)	0.694	(0.251–1.92)	0.482			
current smoker		52 (28.3)	8 (16.7)	0.569	(0.246–1.315)	0.187			
Alcohol consumption [n] (%)									
no alcohol		89 (48.4)	27 (56.3)	1.914	(0.925–3.957)	0.08			
occasionally		31 (16.8)	4 (8.3)	0.502	(0.166–1.513)	0.221			
daily		51 (27.7)	9 (18.8)	0.683	(0.304–1.537)	0.357			
Comorbidities [n] (%)									
Any		161 (87.5)	43 (89.6)	1.229	(0.441–3.421)	0.694			
COPD		8 (4.3)	2 (4.2)	0.957	(0.196–4.658)	0.956			
Renal failure		14 (7.6)	3 (6.3)	0.81	(0.223–2.939)	0.748			
Congestive heart failure		8 (4.3)	1 (2.1)	0.468	(0.057–3.836)	0.479			
Hypertension		88 (47.8)	28 (58.3)	1.527	(0.803–2.904)	0.196			
Cerebrovascular disease		5 (2.7)	3 (6.3)	2.387	(0.55–10.361)	0.246			
Coronary artery disease		21 (11.4)	5 (10.4)	0.903	(0.322–2.532)	0.846			
Diabetes		41 (22.3)	12 (25)	1.163	(0.555–2.436)	0.69			
Deep vein thrombosis		7 (3.8)	3 (6.3)	1.686	(0.419–6.778)	0.462			
Others		48 (26.1)	13 (27.1)	1.045	(0.51–2.139)	0.905			
Previous abdominal surgery [n] (%)		55 (29.9)	11 (22.9)	0.676	(0.329–1.39)	0.287			
Tumor location [n] (%)									
AEG		84 (45.7)	17 (35.4)	0.646	(0.334–1.249)	0.194			
Stomach		82 (44.6)	25 (52.1)	1.339	(0.708–2.531)	0.369			
Diffuse		17 (9.2)	6 (12.5)	1.395	(0.518–3.756)	0.51			
Diagnostics [n] (%)									
Endoscopy		179 (97.3)	48 (100)	433,200,349	(0-)	0.999			
CT		156 (84.8)	45 (93.8)	2.692	(0.782–9.266)	0.116			
MRI		11 (6)	2 (4.2)	0.684	(0.146–3.194)	0.629			
PET		49 (26.6)	11 (22.9)	0.819	(0.388–1.731)	0.601			
Endosonography		116 (63)	25 (52.1)	0.637	(0.336–1.209)	0.168			
Scintigraphy		3 (1.6)	0 (0)	0	(0-)	0.999			
X-ray		79 (42.9)	18 (37.5)	0.797	(0.415–1.532)	0.497			
Sonography		84 (45.7)	15 (31.3)	0.541	(0.275–1.064)	0.075			
Contrast swallow		39 (21.2)	6 (12.5)	0.531	(0.21–1.34)	0.18			
cT Staging [n] (%)									
cT1		3 (1.6)	0 (0)	0	(0-)	0.999	0.532	(0.045–6.252)	0.616
cT2		18 (9.8)	1 (2.1)	0.231	(0.03–1.788)	0.161			
cT3		132 (71.7)	27 (56.3)	0.744	(0.339–1.631)	0.46			
cT4		16 (8.7)	8 (16.7)	2.6	(1.021–6.618)	**0.045**			
cN + Status [n] (%)		131 (71.2)	34 (70.8)	2.024	(0.741–5.528)	0.169			
cM + Status [n] (%)		21 (11.4)	12 (25)	2.514	(1.095–5.776)	**0.03**	17.672	(3.06–102.052)	**0.001**
EUS Staging [n] (%)									
T1:		3 (1.6)	0 (0)	0	(0-)	0.999			
T2:		12 (6.5)	0 (0)	0	(0-)	0.999			
T3:		78 (42.4)	16 (33.3)	1.365	(0.426–4.505)	0.589			
T4:		12 (6.5)	4 (8.3)	1.937	(0.555–6.761)	0.3			
EUS N + Status [n] (%)		46 (25)	9 (18.8)	1.533	(0.505–4.651)	0.451			
WHO grade									
G1		7 (3.8)	0 (0)	0	(0-)	0.999	2.329	(0.343–15.817)	0.387
G2		47 (25.5)	7 (14.6)	0.531	(0.218–1.295)	0.164			
G3		95 (51.6)	30 (62.5)	2.662	(1.099–6.446)	**0.03**			
Barett carcinoma [n] (%)		10 (5.4)	2 (4.2)	0.764	(0.162–3.613)	0.735			
Laurén Classification [n] (%)									
Intestinal		62 (33.7)	6 (12.5)	0.277	(0.105–0.730)	**0.009**	0.909	(0.145–5.691)	0.919
Diffuse, mixed, undifferentiated		63 (34.2)	22 (45.8)	3.608	(1.37–9.504)				
Signet ring cells [n] (%)		34 (18.5)	20 (41.7)	3.246	(1.632–6.458)	**0.001**	6.228	(1.151–33.716)	**0.034**
UICC [n] (%)									
IA		1 (0.5)	0 (0)	0	(0-)	1			
IB		4 (2.2)	0 (0)	0	(0-)	0.999			
II		39 (21.2)	5 (10.4)	0.506	(0.186–1.375)	0.182			
IIIA		80 (43.5)	17 (35.4)	0.894	(0.45–1.778)	0.75			
IIIB		5 (2.7)	1 (2.1)	0.88	(0.1–7.741)	0.908			
IV		52 (28.3)	18 (37.5)	1.941	(0.968–3.893)	0.062			
Time from diagnosis to laparoscopy [days] (IQR)		33 (23–44.7)	30 (21–49)	1.005	(0.992–1.017)	0.455			

Abbreviations: *IQR *interquartile range, *ASA *American Society of Anesthesiologists Classification, *BMI *body mass index, *COPD *chronic obstructive lung disease, *AEG *adenocarcinoma of the esophagogastric junction, *CT *computed tomography, *MRI *magnetic resonance imaging, *PET* positron emission tomography, *EUS *endoscopic ultrasonography, *WHO *World Health Organization, *UICC *Union Internationale Committee Le Cancer Classification

The significant outcomes according to univariate analysis were cT4 stage (OR: 2.6; 95% CI: 1.021 to 6.618; *p* = 0.045), cM-positive stage (OR: 2.514; 95% CI: 1.095 to 5.776; *p* = 0.03) and WHO G3 grade (OR: 2.662; 95% CI: 1.099 to 6.446; *p* = 0.03). Further significant variables according to the univariate analysis were histological evidence of signet ring cells (OR: 3.246; 95% CI: 1.632 to 6.458; *p* = 0.001) and the Lauren type of diffuse, mixed, or undifferentiated carcinoma (OR: 3.608; 95% CI: 1.37 to 9.504; *p* = 0.009).

After multivariate analysis, cM+-category (OR: 17.672; 95% CI: 3.06 to 102.052; *p* = 0.001) and histological evidence of signet ring cells (OR: 6.228; 95% CI: 1.151 to 33.716; *p* = 0.034) were retained as significant factors associated with detection of occult metastasis in patients undergoing SL.

## Discussion

Staging laparoscopy is a sensitive screening method for the detection of peritoneal metastasis and can help to indicate precise therapeutic options for patients with GC or AEG [23]. SL allows for tissue excision and thereby can allow for the detection of additional, occult metastasis, which may remain undetected by other imaging modalities [24, 25]. Therefore, SL is recommended to exclude occult peritoneal metastasis prior to surgery in these patients.

As a consequence, after SL, surgery can be avoided in up to 16.2% of patients with GC [26]. Until now, the occurrence of metastases in patients with GC and AEG has usually led to a palliative treatment intention [27–29]. However, new oncologic therapeutic approaches might change the prognosis of patients with localized and limited resectable metastasis [29, 30]. 

A promising approach for these patients could be neoadjuvant systemic chemotherapy, followed by cytoreductive surgery combined with HIPEC. However, the careful selection of patients with limited peritoneal carcinomatosis is essential for this approach to be successful [18, 21]. Consequently, the current literature emphasizes an individualized and multidisciplinary approach for treating metastatic upper gastrointestinal cancer [31]. The metastatic pattern of gastric cancer is relevant in that the prognosis depends on the extent and location of the metastasis. Distant lymph node metastasis have a better prognosis compared to metastasic burden of a single organ, whereby the latter on the other hand had a better prognosis than multi-organ metastases [32]. 

Knowledge about the exact tumor burden is essential for further therapeutic decision-making and the precise assessment of peritoneal carcinomatosis seems best possible with SL. Additional factors might help to improve risk stratification regarding the occurrence of metastasis in patients with GC and AEG. Therefore, we aimed to identify the risk or protective factors for the occurrence of further, occult metastasis in patients undergoing SL.

Our results indicate that a tumor with signet ring cells is an independent risk factor for the detection of occult metastasis during SL. According to our analysis, patients with signet ring cell carcinomas have a 6-fold higher risk of occult metastasis detection during SL than patients without signet ring cell carcinomas. However, recent literature regarding the prognosis of signet ring cell carcinomas seems to be contradictory. In the early stages of GC, patients with signet ring cell carcinomas appear to have comparably better survival rates, whereas advanced GC containing signet ring cells is associated with poorer survival than is carcinoma without signet ring cells [33]. The survival data of patients after surgery of gastric cancer showed no significant difference in survival between patients with or without signet ring cells for stage I and II. However, a multivariate analysis revealed signet ring cell carcinoma as a significant risk factor for a poor outcome [34]. According to a previous retrospective study, signet ring cells were associated with peritoneal carcinomatosis according to univariate analysis, but not according to multivariate analysis [35]. Similarly, Allen et al. reported that the percentage of signet-ring cell carcinomas was greater among patients with peritoneal metastasis found during SL than among those without occult peritoneal metastasis found during SL. However, the results were not statistically significant [16]. 

The present study emphasizes the importance of these histopathological findings and their relevance for staging procedures. The significantly higher risk of occult metastasis might justify the use of SL independent of the tumor stage and other staging diagnostics if histopathological diagnosis reveals signet ring cells.

Additionally, suspicion of metastasis during CT staging (cM+) was correlated with a 17-fold higher risk of occult metastasis detection during SL. These findings are corroborated by previous publications that identified the presence of more than one metastatic site as a risk factor for peritoneal carcinomatosis [36]. Accordingly, if metastasis is suspected during the course of staging diagnostics, occult metastasis should always be assumed until proven otherwise.

In this context, it should be mentioned that even though the specificity of CT for detecting small peritoneal lesions seems to be quite accurate, the sensitivity remains admittedly deficient, especially for small peritoneal lesions [25, 37, 38]. In a review by Ho et al., various staging methods were compared in terms of sensitivity and specificity to diagnose peritoneal carcinomatosis. In direct comparison, staging laparoscopy showed better results in sensitivity, specificity and also positive predictive value and negative predictive value [38]. However, combining imaging with modern technologies such as deep learning might help to improve the sensitivity of CT up to 87.5%.^39^

For now, the weaknesses of CT diagnostics continue to underscore the important role of SL, which for now is the superior staging method because it offers a good opportunity to reliably diagnose smaller peritoneal metastases in particular [38]. 

### Limitations

This study has several limitations that should be considered when interpreting the results. The retrospective study design has inherent limitations that can impact the quality of our results. The most significant drawback is the potential for selection bias, especially because the indications for staging laparoscopy might have varied over the long period of time during which patients were included.

Another potential limitation of our study might be the inclusion of AEG tumors. In particular, Siewert type I AEGs are usually assumed to originate from the esophagus and are treated accordingly [39]. Epidemiological data on AEG are scarce, with some authors categorizing it as gastric or esophageal cancer depending on the exact anatomical location of the tumor. However, recent publications have identified close molecular analogies between GC and AEG tumors [40]. As a consequence, it is recommended to stage and manage GC and AEG type II or type III similarly [39, 41]. 

## Conclusion

Current guidelines and the literature recommend SL mainly for advanced GC and AEG II and III. However, according to our results the histological diagnosis of signet ring cell carcinoma is an independent risk factor for the occurrence of occult metastasis in patients who undergo SL. Therefore, further evaluation of whether the histological diagnosis of signet ring cells might be considered an independent indication of SL should be performed. In addition, patients with positive cM staging could benefit from extended staging diagnostics, especially SL, as their risk for further, occult metastases is high.

## Electronic supplementary material

Below is the link to the electronic supplementary material.

Supplementary Material 1

## Data Availability

No datasets were generated or analysed during the current study.
