# Supplementary material for: Predictive factors for the detection of occult metastases during staging laparoscopy in patients with gastric carcinoma and adenocarcinoma of the esophagogastric junction
Source: Langenbecks Arch Surg. 2025 Jul 4;410(1):215. doi: 10.1007/s00423-025-03783-9 (PMC12227447; doi:10.1007/s00423-025-03783-9)
Supplement: Supplementary file 1 — Supplementary Material 1 [file 423_2025_3783_MOESM1_ESM.docx]

Supplementary Material:

Supplementary Material Table 1: Location of radiographic metastasis (cM). Distant lymph nodes were defined as lymph nodes other than the regional lymph node stations 1 – 12 and 14v.

|  | Location of radiographic metastasis  n (% of all cM-positive) |
| --- | --- |
| Peritoneum | 13 (39.5) |
| Liver | 6 (18.2) |
| Distant lymph nodes | 11 (33.3) |
| Lung | 1 (3.0) |
| Bone | 2 (6.1) |
